# War injuries and antimicrobial resistance: what impact do multi-resistant pathogens have on the treatment of Ukrainian war- wounded patients?

**DOI:** 10.1007/s00068-026-03116-5

**Published:** 2026-02-23

**Authors:** Sebastian Schreiber, Vakhtang Pirpilashvili, David Osche, Philipp Mörsdorf, Tobias Fritz, Harun Hawi, Sophie E. Müller, Antonius Pizanis, Sören L. Becker, Tim Pohlemann, Emmanouil Liodakis, Marcel Orth

**Affiliations:** 1 Department of Trauma, Hand and Reconstructive Surgery, Saarland University, Homburg, Germany; 2 Institute of Medical Microbiology and Hygiene, Saarland University, Homburg, Germany

**Keywords:** War injuries, Multidrug resistant pathogens, Ukraine, *Klebsiella pneumoniae*, *Pseudomonas aeruginosa*

## Abstract

**Purpose:**

War injuries are characterized by a high incidence of bone and soft tissue infections caused by multidrug-resistant (MDR) pathogens. We analyzed the influence of infections with MDR pathogens on the course and outcome of 17 Ukranian patients within the first three years of surgical therapy.

**Methods:**

To assess the clinical course of Ukrainian war-wounded patients treated in our tertiary care hospital between June 2022 and May 2025, the characteristics of hospital stay, number of operations, bacterial spectrum of wound infections and antibiotic therapies were analyzed retrospectively.

**Results:**

Patients had sustained mostly severe extremity injuries several months (105.3 ± 25.1 days) prior to admission. The average length of hospital stay was 165.6 ± 29.6 days. An average of 13 ± 3 operations were performed per patient during this time. Secondary limb amputation was required in 3/17 patients (18%). MDR bacteria were detected in 11/17 cases (65%). The most common pathogen (in 9/17 patients (53%)) was carbapenem-resistant *Klebsiella pneumoniae*. The most frequently detected carbapenemase was New Delhi metallo-beta-lactamase. Due to multiple resistance, antibiotic therapy with ‘last-resort’ antibiotics such as aztreonam/avibactam or cefiderocol was initiated in 12/17 cases (71%).

**Conclusion:**

MDR pathogens and high rates of carbapenem resistance are commonly detected in war-injured patients. Traumatized patients should be given special importance in an interdisciplinary treatment concept, given the long hospital stays in a foreign country, MDR pathogens, and imminent amputation of a limb. A successful treatment frequently requires the individualized use of novel antibiotics in combination with radical surgical debridement.

**Level of Evidence:**

III, Retrospective/Cohort analysis

## Introduction

War injuries as e.g. gunshot wounds or explosions are high-energy traumas that are characterized by a high incidence of bone injuries in combination with severe soft tissue disruptions [1]. These injuries are often associated with infections caused by multidrug-resistant (MDR) pathogens [2, 3]. Additionally, it is known that migrants, refugees and asylum seekers are in many settings more likely to carry or be infected by MDR bacteria than the host-country population. Antimicrobial resistance (AMR) is thus a major problem, and the most important pathogens include Gram-positive bacteria such as methicillin-resistant *Staphylococcus aureus* and MDR Gram-negative bacteria such as *Klebsiella pneumoniae*, *Escherichia coli*, *Pseudomonas aeruginosa* and *Acinetobacter baumannii* complex. The spread of AMR in such settings is multifaceted and can in part be explained by exposure to conditions that support the emergence of drug resistance (such as living in overcrowded camps) [4]. Infections with AMR represent a global healthcare crisis [5]. In 2019, the estimated global death toll from AMR pathogens was around 1.3 million individuals [5]. The close association between armed conflict, war injuries and infections is a well-recognized problem [6, 7]. Analyses have shown that the bacterial spectrum of wounds changes over time. While low-virulence pathogens tend to predominate in the early phase after trauma, MDR pathogens are often found in later phases of wound healing and their disorders [6, 7]. However, it is frequently very challenging to judge whether the microbiological detection of a specific bacterium in a non-sterile body compartment (e.g. skin) represents mere colonization or true infection.

In line with these findings, the treatment of current Ukrainian war-wounded patients poses a particular challenge to trauma surgeons. In addition to the complex bone and soft tissue defects of the extremities, reconstruction is made more difficult by the increasingly MDR bacterial spectrum. The (sub-)total limb amputation must be considered as ultima ratio [8].

Currently, war injuries are treated in Germany on a post-primary basis as patients are distributed via the clover leaf principle to ensure coordinated evacuation and care. This transfer takes place often weeks or even months after the onset of the primary injury and thereby hampers the surgical treatment. Meanwhile, more than 1,000 patients have been admitted to German treatment centers via this transferal route [8]. A total of 17 Ukrainian patients have been treated in our clinic since February 2022. The aim of the present study was to retrospectively analyze the influence of bone and soft tissue infections on the course and outcome within the first three years of therapy.

## Methods

This retrospective case analysis included a total of 17 patients who were treated in a major trauma center at a university clinic. Ethical approval was waived by the local Ethics Committee in view of the retrospective nature of the study and considering that all the procedures being performed were part of the routine care (permission number: 108/25).

Ukrainian war-injured patients were included, regardless of the pattern of injury, age or gender, type of initial care in the war zone and type of hospitalization in our clinic. All patient data was anonymized for further analysis. The study was conducted from June 2022 to May 2025. All patient data were analyzed retrospectively using the hospital’s internal documentation software SAP i.s.h. med. (SAP i.s.h.med, SAP SE, Walldorf, Germany) and the electronic patient chart Meona (Mesalvo Freiburg GmbH, Freiburg, Germany) as well as the laboratory software (M/LAB, Dorner, Müllheim, Germany).

For the purpose of the study, the following clinical parameters were analyzed:

- type of wounds.
- Injury Severity Score (ISS) at the time of admission.
- average time to admission after trauma (if only the month of trauma was known, the 15th of that month was defined as date of trauma).
- duration of in-patient stay (if admitted to hospital on multiple occasions, or if treated in different departments of the university clinic, the total in-patient stay is calculated by adding together all single admissions).
- number of further out-patient follow-up checks in our clinic.
- number of operations in our clinic.
- bacterial spectrum of wound infections (if any), with “MDR” being defined as non-susceptibility to at least one agent in three or more antimicrobial categories.
- antibiotic treatment (substance, method of application and duration of application; if interrupted, the total duration of intravenous antibiosis was calculated by adding together the individual durations of application).
- clinical outcome within the first three years after treatment (secondary wound closure vs. (partial) limb amputation.
- comparative analyses (MDR-positive vs. MDR-negative) of hospital stay, number of operations and number of amputations.

For microbiological screening analysis of pathogen colonization (including carbapenem resistance testing), swabs were taken in accordance with current World Health Organization (WHO) recommendations upon admission (oral, nasal and rectal). Furthermore, swabs and tissue samples were taken from open wounds and during all surgical procedures throughout the patient’s in-patient stay (based on clinical judgement) and were analyzed subsequently in the microbiological laboratory on campus, using reference diagnostic methods [9]. The individual antibiotic therapy was established in interdisciplinary co-operation with the Institute of Medical Microbiology and Hygiene at the university clinic. Reserve antibiotics were categorized in accordance with the WHO Model List of Essential Medicines (WHO AWaRe [Access, Watch, Reserve] classification, 2023) [10].

All surgical procedures were performed under the responsibility and surveillance of an orthopedic and trauma surgeon according to general surgical guidelines.

Statistical analyses were performed using the SigmaPlot 13.0 software (Systat Software, Incorporated, San José, California, USA). All descriptive data are given as mean ± standard error of the mean (SEM) or as median (range). For comparative analyses, data were first tested for normal distribution (Shapiro–Wilk test) and equal variances (Brown-Forsythe test). An unpaired Student’s t-test was used to compare two experimental groups in case of parametric data. A Mann–Whitney Rank Sum test was performed in case of non-parametric distribution. A p-value ≤ 0.05 was considered to indicate significant differences.

## Results

### Patient characteristics

In total, 14 male and 3 female patients were included (Table 1). The mean age was 41.4 ± 3.2 years. The patient cohort presented with mostly severe extremity injuries following gunshot and blast trauma several months (105.3 ± 25.1 days; unclear in 1 case) prior to admission to our hospital. The mean length of hospital stay after admission to the German trauma center was 165.6 ± 29.6 days. Of interest, 8/17 patients were treated on an intensive care unit (ICU) with an average duration of 6.1 days. Throughout the treatment process, some patients were admitted to hospital on multiple occasions, sometimes in different departments of the treating university clinic. The mean number of further out-patient follow-up checks was 3.5 ± 7.2.

**Table 1:** Baseline characteristics of all patients (n=17)

	gender	age at admission	duration of inpatient stay (d)	cause of trauma	ISS
patient #1	male	21	150	mine explosion	25
patient #2	male	34	354	mine explosion	22
patient #3	male	47	463	mine explosion	13
patient #4	male	33	227	mine explosion	25
patient #5	male	38	105	mine explosion	25
patient #6	male	23	200	mine explosion	29
patient #7	male	53	229	mine explosion	9
patient #8	male	43	14	mine explosion	29
patient #9	male	41	22	gunshot injury	34
patient #10	male	29	55	mine explosion	25
patient #11	female	53	181	gunshot injury	25
patient #12	male	43	234	mine explosion	16
patient #13	female	58	89	bomb explosion	16
patient #14	male	56	239	gunshot injury	13
patient #15	female	65	97	mine explosion	34
patient #16	male	43	149	gunshot injury	41
patient #17	male	24	7	mine explosion	29
mean ± SEM		**41.4 ± 3.2**	**165.6 ± 29.6**		**24.1 ± 2.1**

### Pathogen infection / colonization

Among the 17 patients included in this analysis, two patients had no available full microbiological diagnostics (rectal screening swabs were missing), two patients showed no relevant pathogen detection, and two additional patients had no evidence of MDR pathogens. Consequently, MDR pathogens were identified in 11 of 17 patients (65%), and polymicrobial MDR infections were observed in 10 out of 17 patients (59%). In all 11 cases of MDR pathogen detection in wounds, these were judged as clinically relevant (i.e. there was an infection, not just colonization), meaning there were local signs of infection. In contrast, detections of rectal MDR pathogens were considered as colonization, i.e. not requiring specific treatment.

As revealed by comparative analyses (Table 2), patients who tested positive for MDR pathogens were hospitalized for a significantly longer period of time than those who tested negative (216.64 ± 33.25 vs. 72.00 ± 34.69; *p* = 0.02). Of interest, these patients also underwent surgery significantly more often (17.55 ± 3.86) than patients without MDR pathogen detection (4.67 ± 2.46) (*p* = 0.05 in intergroup analysis). There was no significant difference in the number of amputations (0.18 ± 0.12 vs. 0.17 ± 0.17; *p* = 1.00).

**Table 2:** Comparative analyses of patients with MDR pathogen detection (MDR-positive) and patients without MDR pathogen detection (MDR-negative) evaluating hospital stay (d), number of operations and number of amputations

	MDR-positive	MDR-negative
hospital stay (d)*		
patient #1	150	
patient #2	354	
patient #3	463	
patient #4	227	
patient #5		105
patient #6	200	
patient #7		229
patient #8		14
patient #9		22
patient #10		55
patient #11	181	
patient #12	234	
patient #13	89	
patient #14	239	
patient #15	97	
patient #16	149	
patient #17		7
number of operations*		
patient #1	25	
patient #2	30	
patient #3	32	
patient #4	20	
patient #5		15
patient #6	9	
patient #7		9
patient #8		1
patient #9		0
patient #10		2
patient #11	0	
patient #12	23	
patient #13	7	
patient #14	6	
patient #15	4	
patient #16	37	
patient #17		1
**number of amputations**		
patient #1	0	
patient #2	1	
patient #3	0	
patient #4	0	
patient #5		0
patient #6	0	
patient #7		0
patient #8		0
patient #9		0
patient #10		1
patient #11	0	
patient #12	0	
patient #13	0	
patient #14	1	
patient #15	0	
patient #16	0	
patient #17		0

* = p ≤ 0.05 in intergroup comparison

The most frequently detected carbapenem-resistant organism was *K. pneumoniae*, isolated in 9 patients (53%), followed by MDR *P. aeruginosa* in 7 patients (41%), and *Providencia stuartii* in 6 patients (35%). In two patients, carbapenem-resistant *A. baumannii complex* was identified. MDR *Enterobacterales* with retained susceptibility to carbapenems included *Serratia marcescens*,* K. pneumoniae*,* E. coli*, and *Proteus mirabilis*.

The most frequently identified carbapenemases in *Enterobacterales* were New Delhi-beta-lactamase (NDM) (in some isolates co-produced with additional carbapenemases), followed by OXA-48 and *K. pneumoniae* carbapenemase (KPC) as revealed by microbiological analyses (Table 3). Imipinem hydrolyzing metallo-beta-lactamases (IMP)-type carbapenemases were detected in *P. aeruginosa*, while OXA-23 was identified in *A. baumanii complex* isolates.

**Table 3:** Carbapenemase production among *Enterobacterales* with carbapenem resistance detected in specimens stemming from 17 Ukrainian patients with war injuries (^1^ Those included kidney punctures, skin swabs, and stool samples)

Body site	NDM	KPC	OXA-48	Multiple carbapenemases
Rectal screening	E. coli, P. stuartii (2 patients), K. pneumoniae	E. coli, K. pneumoniae, P. stuartii	K. pneumoniae	**NDM + OXA-48**: K.pneumoniae (2 patients) **NDM + KPC**: K. pneumoniae
Wound swab/ biopsy	K. pneumoniae (5 patients), E. coli, P. stuartii (4 patients), P. mirabilis	K. pneumoniae	E. coli, K. pneumoniae	**NDM + KPC**: K. pneumoniae **NDM + OXA-48**: E. cloacae complex, E. coli, K. pneumoniae (2 patients)
Blood culture				**NDM + OXA-48**: K. pneumoniae
Urine	K. pneumoniae, P. stuartii		K. pneumoniae	**NDM + OXA-48**: K. pneumoniae
Other^1^	K. pneumoniae, P. stuartii	K. pneumoniae	K. pneumoniae	**NDM + OXA-48**: K. pneumoniae

### Antibiotic therapy

In total, 25 different intravenous antibiotics were used (Table 4). After the initial empirical antibiotic treatment, therapy was adjusted in collaboration with infectious disease consultancies provided by the hospital’s clinical microbiologists, according to the individual antimicrobial susceptibility profile. Intravenous antibiotic treatment was initiated in 15/17 patients. On average, intravenous antibiotics were administered for 71 (± 16.1) days. Remarkably, one patient was given intravenous antibiotics for a total of 253 days. It should be noted that the intravenous antibiotic treatment was sometimes interrupted due to associated side effects such as nephrotoxicity or diarrhoea, but was resumed during the course of treatment. Due to the large number of resistances, antibiotic therapy was initiated in 12/17 cases (71%) with reserve antibiotics with activity against carbapenemase-producing bacteria, e.g. ceftazidime/avibactam, cefiderocol and aztreonam/avibactam. According to the WHO AWaRe list, 8 “access-“, 11 “watch-“ and 6 “reserve- antibiotics” were used (Table 4). Five patients underwent repeated local antibiotic therapy (PALACOS R + G^®^ [Heraeus GmbH, Hanau, Germany] or GENTA-COLL^®^ resorb [Resorba Medical GmbH, Nürnberg, Germany]) with gentamicin.

**Table 4:** Intravenous antibiotic administered to patients with war injuries; Patients received either antibiotics according to the WHO AWaRe-classification 2023 of the reserve group (^1^), watch group (^2^) or access group (^3^)

intravenous antibiotic	administered in x/17 patients
ceftazidim/avibactam (+ aztreonam)^1^	7
colistin^1^	6
cefiderocol^1^	3
fosfomycin^1^	3
daptomycin^1^	1
meropenem^2^	8
vancomycin^2^	8
piperacillin/tazobactam^2^	5
tigecyclin^2^	4
cefuroxim^2^	3
levofloxacin^2^	2
erythromycin^2^	1
ciprofloxacin^2^	1
ceftriaxon^2^	1
imipenem/cilastatin^2^	1
clindamycin^2^	1
ampicillin/sulbactam^3^	3
ampicillin^3^	3
flucloxacillin^3^	2
cotrimoxazol^3^	2
metronidazol^3^	2
tetracycline^3^	1
cefazolin^3^	1
amikacin^3^	1

### Surgical therapy

All patients had more than one war injury with an average ISS of 24.1 ± 2.1. The initial injury patterns involved the femur (including the hip joint) in 9/17 cases, the lower leg in 3/17 cases, the pelvis in 4/17 cases, the upper extremity (scapula, humerus, metacarpals) in 5/17 cases, the abdomen including the spine in 2/17 cases and the bones of the skull (orbital bone, ethmoid bone, temporal bone, sphenoid bone) in 1/17 cases. As an average, 13 **±** 3 operations were performed per patient during this period. All operations were performed under the responsibility of a qualified trauma surgeon. Repeated local debridement with additional negative pressure wound therapy was performed in 71% (12/17) of the patients, and secondary (partial) amputation of a limb was required in 18% of the war-injured patients (Table 5). In only 1/3 cases was the criterion for amputation ‘life before limb’. In another case, there was an infected non-union in the distal femur that could not be surgically reconstructed. In the last case, insufficient soft tissue coverage and fistula formation necessitated a revision amputation (after traumatic amputation). In 2 cases, definitive treatment was either provided by the neurosurgery or ophthalmology department. Severe trauma-associated nerve lesions (tibial, peroneal and sciatic nerves) were found in 2/17 cases. Two patients (11.8%) were transferred to another hospital for further treatment. Twelve patients (70.6%) were discharged to their homes, 3/17 patients (17.6%) to rehabilitation centers. None of the patients died from these serious injuries. To the best of our knowledge, none of the patients returned to Ukraine.

**Table 5:** Overview of surgical treatments during the in-patient stay of the war injured patients. Frequency of operations in total as well as the necessity of a vacuseal (VAC) therapy and (partial) amputation of a limb is given

	number of operations performed	VAC-therapy (yes/no)	(partial) amputation of a limb in domo (yes/no)
patient #1	25	yes	no
patient #2	30	yes	yes
patient #3	32	yes	no
patient #4	20	yes	no
patient #5	15	yes	no
patient #6	9	yes	no
patient #7	9	yes	no
patient #8	1	no	no
patient #9	0	no	no
patient #10	2	no	yes
patient #11	0	no	no
patient #12	23	yes	no
patient #13	7	yes	no
patient #14	6	yes	yes
patient #15	4	yes	no
patient #16	37	no	no
patient #17	1	yes	no
mean ± SEM / yes: no (%)	13 ± 3	11:6 (65%)	3:14 (18%)

### Multidisciplinary treatment and therapy-related complications

Patients were treated on ICU for an average of 6 **±** 3 days, with one patient receiving intensive care for a total of 49 days. As part of a multidisciplinary therapy concept and apart from the infectious disease consultations, a total of 18 different departments at our university hospital were consulted overall, including anaesthesiology (consulted in 13/17 patients), psychiatry (10/17), urology (6/17), general surgery (5/17), ophthalmology (4/17), nephrology (4/17), neurology (3/17), cranio- maxillofacial surgery (3/17), neurosurgery (3/17), cardiology (3/17), gastroenterology (3/17), thoracic surgery (2/17), orthopaedics (2/17), haematology (1), dentistry (1/17), pneumology (1/17), haemostaseology (1/17) and dermatology (1/17). On average, 4 different departments were consulted per patient. Three out of 17 patients suffered from sepsis during the course of their inpatient stay, resulting in consecutive organ damage. Table 6 provides an overview of therapy-associated complications and their frequency. Although an antibiotic treatment regimen tailored to the individual resistance patterns was employed in all patients, wound healing disorders/fistula formations/stump infections were found in 6/17 patients (35,3%), thus emphasizing the complexity of treating MDR bacteria. Of interest, although a psychiatric consultation was carried out in 59%, only three of them received a psychiatric diagnosis (adaptation disorder/moderately severe depressive episode).

**Table 6:** Therapy-associated complications during the treatment and its incidence in the patient cohort

complication	x/17 patients affected
wound healing disorder/fistula formation/stump infection	6
hypokalaemia requiring intensive care	4
sepsis	3
antibiotic-associated renal damage	3
postoperative haemorrhage anaemia	3
thrombosis / pulmonary artery embolism	3
osteomyelitis	2
antibiotic-associated diarrhoea/gastroparesis	2
cachexia /refeeding syndrome	2
adaptation disorder	2
infection with clostridium difficile	1
moderately severe depressive episode	1
knee joint empyema	1
hip joint empyema	1

### Case description

**Figure 1:**
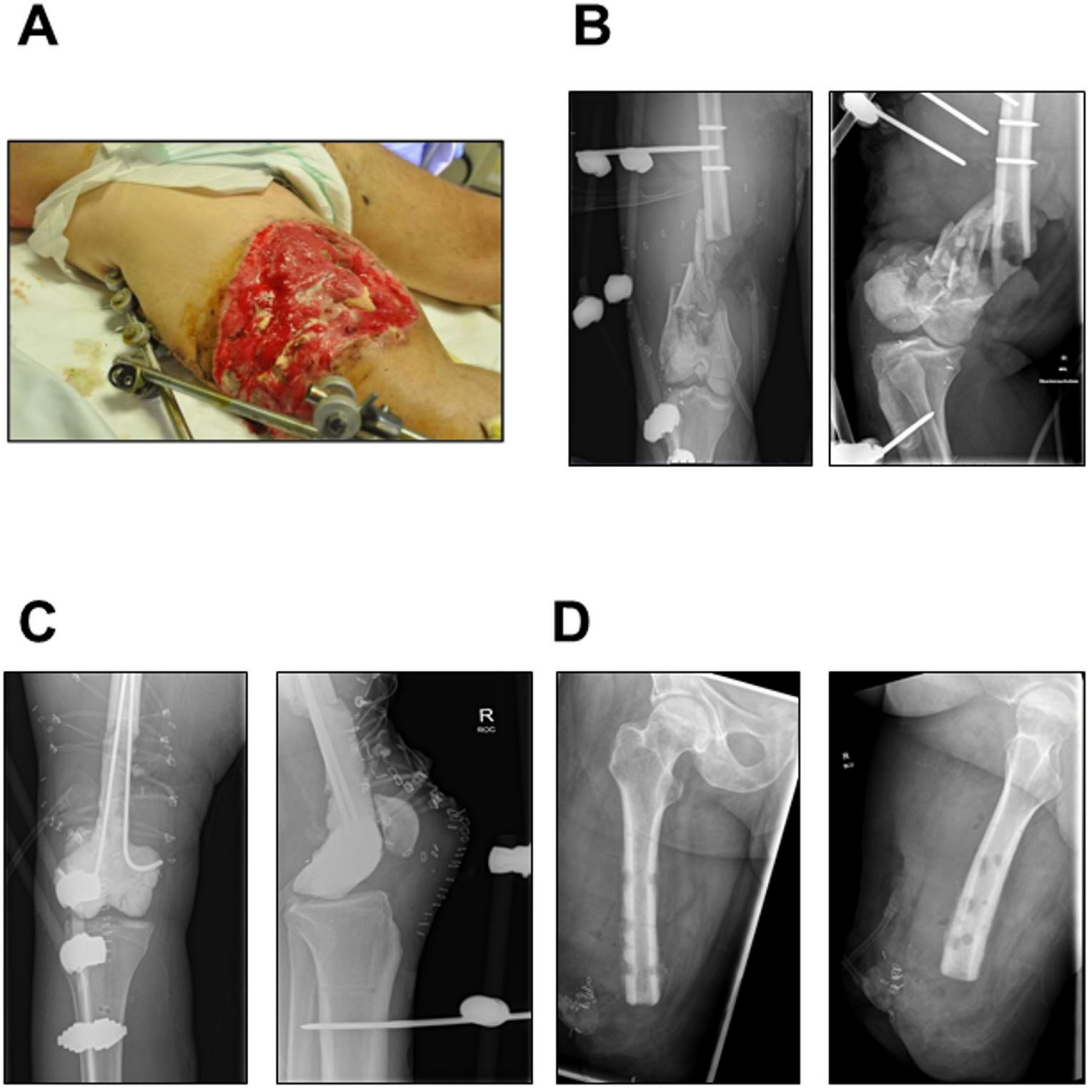
Fig. 1 (**A**) Clinical wound situation of the right lower extremity on the day of admission to our hospital. (**B**) Preoperative a.p. (anterior-posterior) (left) and lateral (right) x-ray of the right knee on the day of admission to our hospital. (**C**) Postoperative a.p. (left) and lateral (right) x-ray of the right knee following treatment with a PALACOS^®^ cement spacer and re-osteosynthesis using an external fixator and wire osteosynthesis. (**D**) Postoperative a.p. (left) and lateral (right) x-ray after major amputation of the right lower extremity. All images from our own hospital

A 56-year-old male patient sustained multiple injuries following a rocket blast and received initial treatment in Ukraine. The medical history documentation was incomplete. Upon admission to our clinic (60 days after trauma), he presented with severe abdominal trauma and a third-degree open distal femoral fracture (ISS 13), type 33C3 according to the Arbeitsgemeinschaft für Osteosynthesefragen (AO) classification system (Fig. 1A, B). According to the enclosed documents, the initial treatment included a longitudinal laparotomy, a partial bowel resection and the application of an external fixator to the femur. The primary treatment strategy aimed to preserve the extremity. With this in mind, radical surgical debridement and placement of an antibiotic-coated spacer (PALACOS^®^) were first performed, after which the construct was stabilized with two wires (Fig. 1C). Initial microbiological wound swabs revealed a polymicrobial colonization/infection with *P. stuartii* (NDM carbapenemase), *K. pneumoniae* (OXA-48 and NDM carbapenemase), and *P. mirabilis* (extended-spectrum beta-lactamase, ESBL). Additionally, *E. coli* (ESBL) and *Morganella morganii* (ESBL) were detected in rectal screening swabs. In line with the current guidelines on the treatment of MDR pathogens (e.g. those put forth by the Infectious Diseases Society of America, IDSA) and in accordance with clinical microbiology consultancies, the patient received a number of antibiotics during his stay, including beta-lactams (e.g. ampicillin/sulbactam, piperacillicin/tazobactam, meropenem, ceftazidime/avibactam + aztreonam), glycylcyclines (i.e. tigecycline), colistin, fosfomycin and metronidazole. Yet, the patient developed a septic shock (with consecutive treatment on ICU for 15 days), accompanied by severe hypocalaemic alkalosis and diarrhoea with secondary gastroparesis, which required several therapeutic adjustments. Blood cultures became positive for carbapenem-resistant *K. pneumoniae* and the yeast *Candida albicans*, and subsequently for MDR *P. aeruginosa*. Although the initial aim had been to preserve the limb, a major amputation of the right lower limb above the knee joint was now being performed following the principle ‘life before limb’ (Fig. 1D). Given the complexity of the case, consultations were sought from the departments of general surgery, psychiatry, gastroenterology, ophthalmology, nephrology, anaesthesia and urology. A moderate depressive episode was diagnosed. The patient was also hospitalized intermittently in the gastroenterology department due to a severe *Clostridioides difficile* infection. Weeks after being discharged from hospital, the patient was admitted again with a silent infection of the amputation stump from which carbapenem-resistant *K. pneumoniae* was isolated. The infection was adequately treated with anti-infective therapy involving colistin and vacuum-assisted closure therapy. The patient has not returned to our clinic since. The patient was hospitalized for a total of 239 days and underwent 6 operations in total.

## Discussion

The results of the present study emphasize the challenges in treating war-wounded patients due to the increasing prevalence of AMR in this very specific patient cohort.

In a recent study, Kovalchuck et al. analyzed the temporal evolution of bacterial species and their AMR in wound infections of Ukrainian war-wounded patients from 2014 to 2023 [7]. Their data revealed a predominance of Gram-negative bacteria with *A. baumannii* complex (35.7%) being the most common, followed by *K. pneumoniae* (20.7%) and *P. aeruginosa* (14.9%). Of interest, Kovalchuk et al. found that the occurrence of *P. aeruginosa* decreased from 29.6% in 2020 to 14.9% in 2023, accompanied by an increase in the frequency of *A. baumannii* complex from 29.6% to 35.7%. In the past, MDR Gram-negative pathogens were found in the majority of cases [6, 7]. In contrast to the aforementioned studies, *A. baumannii* complex was rarely detected in our cohort, with *K. pneumoniae* being the most prevalent pathogen. These differences may be due to the different patient populations or patient number. While Murray et al. [6] primarily examined US military conflicts since World War I, the present data are limited to Ukrainian war casualties. In addition, different climatic and geographical conditions play a role in addition to the predominant local pathogen spectrum. Accordingly, the predominant pathogens in war wounded patients can only be predicted to a limited extent due to the many influencing factors. In line with these different results, Granata et al. found, that war injuries present with a large spectrum of MDR infections that could potentially spread [11]. Therefore, infection control in healthcare facilities within conflict zones and proper antimicrobial stewardship are crucial for direct treatment as well as later therapeutic strategies beyond the conflict area.

The high number of MDR pathogens and antibiotic drugs in patients of the present study illustrate the complicating influence of AMR on the course of treatment. Of interest, an infection with MDR pathogens resulted in a significant higher number of operations and a longer hospitalization as revealed by comparative analyses. The antiinfective treatment becomes even more complex in patients, who present with polymicrobial MDR infection. In this study, one patient exhibited 8 different MDR pathogens and received a total of 10 different antibiotics during his stay. Radical germ eradication could be achieved and the wounds closed secondarily. Following temporary oral administration, the anti-infective therapy could be completed prior to discharge. It is, however, of importance to note that MDR pathogens are by no means necessarily more virulent than non-MDR pathogens, and the distinction between mere colonization and true infection can be extremely challenging. Hence, infectious disease consultations is frequently warranted. However, if local signs of inflammation such as rubor, calor, dolor, tumor or functio laesa occur alongside the detection of a pathogen, it can be assumed that an infection is present.

### Antibiotic therapy

The global AMR problem has significantly increased due to the more frequent detection of MDR Gram-negative bacteria, and in particular carbapenem-resistant and carbapenemase-producing isolates, which were also commonly found in this study. While carbapenems such as meropenem are frequently active in the presence of other resistance mechanisms, carbapenem-resistant *Enterobacterales* (such as *E. coli* and *K. pneumoniae*) and non-fermentative bacteria (e.g. *P. aeruginosa*) require a more refined approach, depending on the detected type of carbapenemase. While OXA-48 and KPC carbapenemases were more frequently found in Germany in previous years, a sharp increase in NDM-producing isolates has been noted since 2022, which is also frequently observed in war-wounded patients from Ukraine [12]. While drugs such as ceftazidime/avibactam are frequently active against OXA-48 and KPC [13], metallo-beta-lactamases such as NDM are more resistant and require other treatment options such as aztreonam/avibactam or cefiderocol and, in some instances, colistin. The rapidly changing epidemiology of the predominant carbapenemase type, challenges underlying the reproducible conduct of antimicrobial resistance testing for some of these novel antibiotics, as well as the limited availability and the high cost of these antibiotics pose significant threats to a successful treatment. International guidelines such as those put forth by the European Society of Clinical Microbiology and Infectious Diseases (ESCMID) [14] or the IDSA [15] provide guidance, but these recommendations are rapidly evolving and there is a lack of high-quality comparative data on the efficacy of the different antibiotics for complex skin and soft tissue infections, bone infections and bacteremia. Hence, a close collaboration between clinical microbiology/infectious diseases and the treating surgical departments as well as early screening for the presence of MDR pathogens in patients from high-risk areas are urgently required.

### Surgical therapy

Surgical therapy, along with anti-infective treatment, is of paramount importance in the overall approach. The heterogeneous nature of the injury patterns results in limited comparability within the cohort. To address this issue, the ISS was calculated retrospectively for every patient at the time of admission. Despite complex osseous destructions, these injuries often involve large soft tissue defects. A multidisciplinary consensus statement has summarized recommendations for the initial debridement of war wounds to the extremities [16]. The consecutive need for radical surgical debridement mostly results in large bone defects. Various surgical procedures have been developed over time to treat such defects. In addition to definitive treatment with an external (ring) fixator, options include segment resection with subsequent callus distraction (internal or external distraction techniques), interposition of a vascularized autologous bone transplant or augmentation by use of osteoconductive allogenic or non-osseous grafts [8]. Moreover, Masquelet’s induced membrane technique is a promising and well-established procedure for restoring continuity of the affected limb [17–19]. It is not uncommon to find scattered foreign bodies such as steel bullets and shrapnel in the wounds. Contaminated clothing and bone fragments are also found in the soft tissues [2]. Because of the known contamination and the increased risk of infection, immediate primary closure is contraindicated. Furthermore, wound infections with multi-resistant pathogens are an additional challenge in surgical treatment. Following evaluation of the microbiological findings, no evidence of surgical site infection was found in any of the 17 cases. During follow-up examinations after discharge, new pathogens were identified in a few cases. Nevertheless, we consider surgical site infection to be unlikely but it is not possible to rule it out with absolute certainty. The outcome of infected and non-infected wounds was compared in a cohort study involving 457 Syrian war wounded [20]. Of interest, there were significantly more amputations (22% vs. 9%), longer hospital stays (55 days vs. 35 days) and more operations (12 vs. 5) in the wound infection group [20]. These results are consistent with the findings of the present study (amputation rate: 18%, mean duration of hospital stay: 165.6 ± 29.6 days, mean number of operations: 13 ± 3), emphasizing the complex nature of infected wounds and their interdisciplinary treatment.

Several studies have demonstrated the benefits of a negative pressure wound therapy in war wounded patients [21–23]. Both, the time to secondary wound closure and the number of secondary infections can be effectively reduced by applying vacuum-assisting therapies [21–23]. Although, negative pressure wound therapy was used in 65% of cases in the present patient cohort, the number of operations per patient (13 ± 3), the hospital stay (165.6 ± 29.6 days) and the fact that a secondary (partial) amputation of a limb had to be performed in 18% of the patient collective (‘life before limb’) remained high and may mitigate these presumed advantages. Accordingly, new wound therapies beyond negative pressure wound therapies may be useful to reduce the risk of wound infections, especially in a vulnerable patient cohort such as polytraumatized war injured patients. Concomitant injuries like nerve lesions can also prolong the rehabilitation process. Nevertheless, secondary wound closure and discharge to a self-determined life was achieved in 6/17 (35%) patients at the end of the study period. Apart from these general aspects of surgical wound care, however, it is not possible to derive a universally applicable algorithm from this cohort. Surgical treatment is usually decided on a case-by-case basis, taking the patient’s overall condition into account.

### Preparation for the worst-case scenario

To prepare for potential crises and conflicts, the political framework must be in place to ensure the population receives adequate medical care. The German Bundeswehr is making a pivotal contribution to providing relevant infrastructure and resources (OPLAN DEU). The DGU TNWs play a significant role in the distribution and care of (Ukrainian) war wounded patients, which are organized efficiently via the joint situation center (Gemeinsames Lagezentrum, GMLZ) and the cloverleaf structure [24].

In addition, it is just as important to provide surgeons with specialized training in these injury patterns as it is to implement antibiotic stewardship [11, 16]. These preparatory measures should primarily aim to enable a prompt and appropriate response to acute changes in the situation.

### Limitations

Rectal screening swabs were not performed on two patients. This could have resulted in MDR pathogen colonization being missed, which is a limitation of this study. Only documented data upon arrival and during treatment in Germany could be included in the present study. War injured patients may have been undergone further treatment in the conflict zones. Due to a lack of documentation, however, some information may be inaccurate or incomplete. Additionally, the ISS is typically calculated when a patient with multiple trauma is first diagnosed and treated. Some of the patients included in this study did not arrive at our clinic until several weeks after their injury. Furthermore, some of the medical documentation was missing. Therefore, the ISS values calculated are as accurate as possible from the existing case documentation. Moreover, not all psychiatric consultations were attended by a professional interpreter. Therefore, it is possible that the psychiatric diagnoses in this study are lower than they should be.

### Summary

The present data demonstrates that despite multidisciplinary high-end treatment approaches, close collaboration with institutes of other specialties and the availability of state-of-the-art surgical techniques, the increasing development of microbial resistance poses a major challenge to the overall management of patients with war injuries. In view of the expected increase in AMR, special importance should be attached to thorough microbiological resistance testing in (Ukrainian) war-wounded patients, including prompt testing for carbapenemase types and for recently introduced antibiotics such as aztreonam/avibactam and cefiderocol. Furthermore, action is needed to improve infection prevention, antimicrobial stewardship and AMR awareness in line with the 2015 WHO Global Action Plan and the findings of the fourth report of the WHO Global Evidence Review on Health Migration [25, 26]. The high MDR detection rates call for a compulsory rectal screening for MDR carriage and preventive single room isolation of war-injured individuals until microbiological screening results are available. In view of long hospital stays in a foreign country, prolonged isolation and the (imminent) amputation of a limb, traumatized patients should be given special importance in an interdisciplinary treatment concept. This study raises awareness of the often complication-prone course of severe war injuries. Therefore, trauma surgeons will have to lead these patients throughout this multidisciplinary treatment and may need to undergo special preparation for war injured patient cohorts.

In summary, the number of MDR pathogens is increasing at the global level. Radical surgical debridement of war injured patients is often required in the interdisciplinary management with MDR pathogens. Tailored treatment approaches using novel anti-infectives are essential for successful treatment.

## Data Availability

The data that support the findings of this study are not openly available due to reasons of sensitivity and are available from the corresponding author upon reasonable request.
